# Notes and News

**Published:** 1903-08-08

**Authors:** 


### Notes and News.

To mark the visit of their Majesties to Dublin, Lord Iveagh has given a sum of £50,000 to the King to be distributed among the Dublin hospitals, Protestant and Catholic alike, including the National Hospital for Consumption, co. Wicklow.

Owing to the large number of accidents occurring among dock labourers the Home Secretary is taking the necessary steps to include the occupation in the schedule of dangerous trades, and so bring it under the provisions of the Factory Act, 1901, relating thereto.

Mr. Frederick Howard Marsh, F.R.C.S., surgeon to St. Bartholomew's Hospital, has been elected to the Chair of Surgery in the University of Cambridge, which has been vacant since the death of Sir George Humphry in 1896. Apparently there had been some discussion as to whether the Chair in Surgery should be abolished altogether at Cambridge; having decided to retain it, the University are to be congratulated on having found such a worthy successor to Sir George Humphry.

It would appear that the inspection of bakehouses, of which so much has been said of late, is not enough to protect the consumers against possible evils. Not only the bakehouse but also the baker should be kept under supervision. Dr. Dudfield, the Medical Officer of Health for Kensington, reports that in the course of his inquiries he found one baker at work while he was suffering from very active secondary syphilis, another had facial erysipelas, and a third epithelial cancer. One need hardly comment on the offensiveness of persons suffering from any skin disease handling the bread we eat, and even in machine-made bread a certain amount of handling can scarcely be avoided, although that method is a great improvement on the old ways.

The Drapers' Company has promised a donation of £4,000 to the Tottenham Hospital on condition that the authorities of that institution raise £3,000 for much-needed improvements.

Upwards of £40,000 have been bequeathed by the late Miss Mary Thompson to be divided between the London Hospital, the Surgical Aid Society, Salisbury Square, the Royal Hospital for Diseases of the Chest, City Road, the London City Mission, and the British and Foreign Bible Society.

The Fishmongers' Company have recently been taking steps with a view to restoring public confidence in oysters as articles of food. For this purpose they have issued a statement in which they report that "they have inspected and caused samples of oysters to be taken from many fisheries on different parts of the coast whence oysters are sent to the London market and submitted them for examination to their official analyst, one of the leading bacteriologists of this country. As a result, certain beds, pits, and layings were reported so far contaminated as likely to be a source of danger to the public. These have now been closed, and no oysters from these places will knowingly be allowed to be sold or placed on the market for sale until they have been proved to be safe and wholesome, and their insanitary surroundings satisfactorily remedied." This is, of course, a step in the right direction, but it is more than a pity that the investigations were not extended to all the sources of supply, so that the consumer might be in a position of comparative certainty with regard to the wholesomeness of the oysters he consumes.

Experts in sociology are so generally agreed that emigration offers the best chance for lifting pauper children out of the pauper class that it is a pity that there should be a prejudice against it among these children and their friends. The Lambeth guardians recently published the names of 91 children whom they proposed to emigrate to Canada. As soon as the posters containing the names had been read, 10 children were reclaimed by their friends, 17 children themselves refused to emigrate, and the parents of some others refused their consent. The Church of England Waifs and Strays Society undertook the care of a considerable number, meaning also to emigrate them, but the same difficulties pursued their efforts, and in the end only five out of the 91 children were sent to Canada. It is probable that in the case of those who were claimed by friends on the publication of the names, the appearance of the poster was an indication to the relations of the children that they were now of an age to contribute to their own support and probably to that of their kindred. Parents, as well as other relatives, who are quite willing to let the State bear the burden of bringing up children very often claim them as soon as they think that something can be got from them. The pity of it is that they thus bring them back to the very environment which made them paupers in the beginning, and defeats the well-intentioned efforts of the State to raise their status.

